# How to Optimize ECLS Results beyond Ventricular Unloading: From ECMO to CentriMag^®^ eVAD

**DOI:** 10.3390/jcm11154605

**Published:** 2022-08-07

**Authors:** Vincenzo Tarzia, Lorenzo Bagozzi, Matteo Ponzoni, Giacomo Bortolussi, Giulio Folino, Roberto Bianco, Fabio Zanella, Tomaso Bottio, Gino Gerosa

**Affiliations:** Cardiac Surgery, Department of Cardiac, Thoracic and Vascular Sciences and Public Health, University of Padova, 35122 Padova, Italy

**Keywords:** ECMO, left ventricle unloading, extracorporeal VAD, LVAD

## Abstract

CentriMag^®^ extracorporeal VAD support could represent a more physiological choice than conventional ECMO in primary cardiogenic shock. We therefore evaluated the outcome of patients with primary cardiogenic shock who were supported with CentriMag^®^ extracorporeal VAD implantation versus conventional ECMO. We retrospectively reviewed all extracorporeal life supports implanted for primary cardiogenic shock between January 2009 and December 2018 at our institution. Among 212 patients, 143 cases (67%) were treated exclusively with ECMO (Group 1) and 69 cases (33%) with extracorporeal VAD implantation (Group 2, 48 of whom as conversion of ECMO). ECLS mean duration was 8.37 ± 8.43 days in Group 1 and 14.25 ± 10.84 days in Group 2 (*p* = 0.001), while the mean rates of the highest predicted flow were 61.21 ± 16.01% and 79.49 ± 18.42% (*p* = 0.001), respectively. Increasing mechanical support flow was related to in-hospital mortality and overall mortality in Group 1 (HR 11.36, CI 95%: 2.19–44.20), but not in Group 2 (HR 1.48, CI 95%: 0.32–6.80). High-flow ECMO patients had lower survival with respect to high-flow extracorporeal VAD patients (*p* = 0.027). In the setting of high-flow mechanical circulatory support, CentriMag^®^ extracorporeal VAD optimized patient survival, granting long-term assistance and physiological circulation patterns.

## 1. Introduction

Veno-arterial extracorporeal membrane oxygenation (VA-ECMO) is widely used to support circulation during severe heart failure refractory to standard medical treatment [1,2,3]. This mode of support carries intrinsic drawbacks despite its advantages. VA-ECMO is associated with increased left ventricular (LV) afterload, particularly when *high-flow* support is required [4]. During full ECMO support, the LV is unable to eject blood and the aortic valve fails to open, which may lead to LV overload. This leads to increased LV wall stress and myocardial oxygen consumption, raising hydrostatic pressure in the lung circulation [4,5].

In such cases, adequate LV decompression may be an important factor associated with better patient outcomes [6]. Several techniques are used to accomplish LV decompression during high-flow ECMO, such as apical LV venting, IABP, Impella^®^ system, and conversion to CentriMag^®^ extracorporeal temporary ventricular assist device (eVAD) [7]. Among these, the latter is the only one able to provide full circulatory support, based on a more physiological pattern [8].

The aim of this study was to evaluate the optimal extracorporeal life support (ECLS) configuration in primary cardiogenic shock, tailored to patient’s hemodynamic status and needed mechanical support. In particular, we sought to assess in which kind of patients the conversion from ECMO to CentriMag^®^ eVAD is effective to reduce in-hospital and overall mortality.

## 2. Materials and Methods

### 2.1. Study Population

Between January 2009 and December 2018, a total of 871 patients were implanted with ECLS at Padova University Hospital. We analyzed 212 patients implanted with ECLS for treatment of primary cardiogenic shock refractory to optimized medical therapy and/or IABP. According to ELSO definitions, we mean by ECLS every type of extracorporeal mechanical circulatory support, namely both veno-arterial ECMO and extracorporeal ventricular assist devices [9].

ECLS implantations for postcardiotomy syndromes or respiratory support, as well as the pediatric population (<18 years), were excluded. Written informed consent was obtained from all patients, and the study was approved by our institutional Ethics Committee (Protocol 39706, June 2022). Follow-up data completeness was 100%.

At the time of ECLS implantation, 191 patients (90%) were supported by ECMO. Of these, 116 patients (61%) were implanted using a percutaneous femoral technique at the bedside; 47 patients (25%) underwent ECMO implantation during cardio-pulmonary resuscitation.

**Figure 1:**
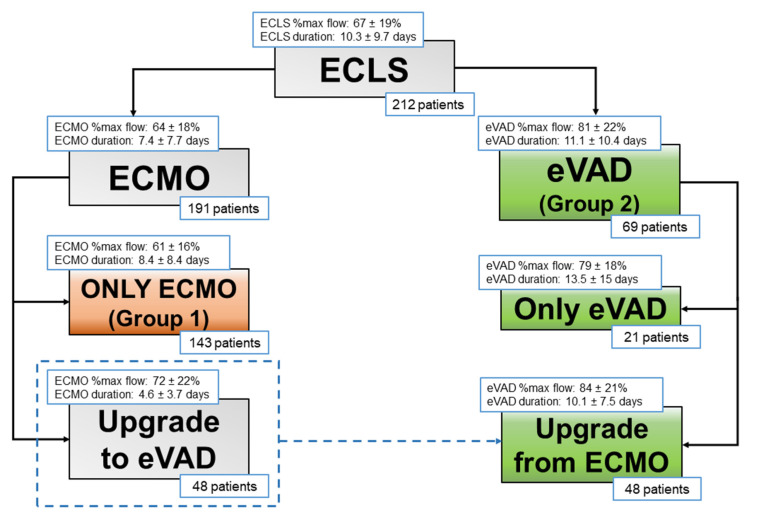
Study population groups.

Most patients were kept on ECMO support (143 patients, 67%, Group 1), while 69 patients (33%, Group 2) were treated with CentriMag^®^ eVAD, as first-line support (21 patients) or as conversion from ECMO (48 patients) (Figure 1).

### 2.2. ECLS Devices

The system used for ECMO was the PLS^®^ (Maquet, Cardiopulmonary AG, Hirrlingen, Germany); this is a portable “all-in-one” design for mechanical circulatory assistance including an oxygenator (Quadrox D), a centrifugal pump (Rotaflow), heparin-coated tubes and an optional heat-exchanger. For eVAD, we used the Levitronix CentriMag^®^ mechanical circulatory support system (Thoratec, Pleasanton, CA, USA) with a free-floating magnetically levitated rotor pump, assembled with an oxygenator and a heat-exchanger if needed. The decision to adjunct an oxygenator is based on the clinical status of the patient, chest X-rays, arterial blood gas exchange analysis, and needed fractional inspired oxygen percentage during ECMO to achieve adequate blood gas balance.

### 2.3. ECLS Placement

**Figure 2:**
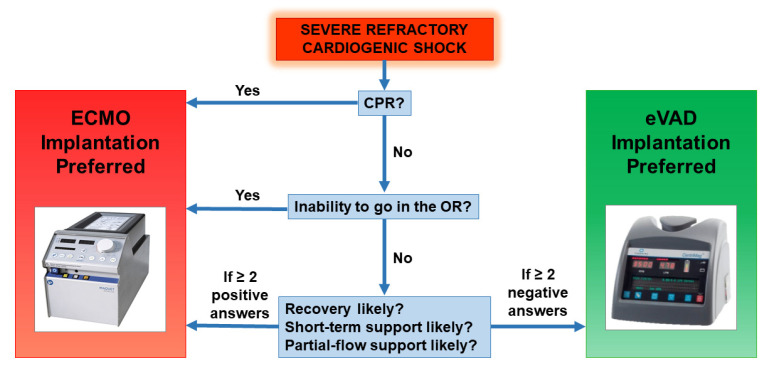
Decision-making flow chart for ECLS instauration and subsequent management.

Every patient affected by cardiogenic shock undergoes a clinical and echocardiographic evaluation to assess which is the best initial treatment option. The ECMO configuration is chosen in the following settings: implantation during cardio-pulmonary resuscitation, inability to transport the patient in the operating room, likely need for short ant partial-flow support, high probability of ventricular recovery. Other cases are suitable for direct eVAD implantation. Our decision-making flow chart is provided in Figure 2.

Whenever feasible, we opted to implant ECMO at the bedside with the patient awake and breathing independently. When this strategy was unsuccessful or contraindicated, the patient underwent surgical implantation.

The implantation of the CentriMag^®^ eVAD was always performed in the operating room. For left VAD positioning, we utilized a left mini-thoracotomy in the fifth intercostal space for direct LV apex cannulation, after placing a double-crossed purse-string suture. The return cannula was placed in the femoral or subclavian artery. In the case of a biventricular VAD implantation, we performed a full sternotomy or double mini-thoracotomy. For right VAD support we adopted a femoral vein main pulmonary artery configuration.

### 2.4. Management of ECLS

After ECLS implementation, the majority of patients need full theoretical flow support (calculated as BSA × 2.4 L/minute), totally replacing the cardiac function.

Afterward, the reappearance of arterial pulsatility may be observed: this is a sign of initial recovery of cardiac function and LV unloading. During this phase, we always try to wean patients from mechanical ventilation, achieving extubation if possible. In the meanwhile, ECMO support is decreased (*partial-flow* support), monitoring pulsatility, median arterial pressure, and organ perfusion (by means of lactate clearance, central venous oxygen saturation, and urine output when appropriate). We maintain a medium dose of inotropic therapy, in order to support myocardial contractility to grant the opening of the aortic valve, thus eliminating the need for artificial venting. This concept of *partial-flow* support allows unloading while maintaining organ perfusion.

Conversely, when *full-flow* support is needed for more than a few days to achieve adequate organ perfusion, in the ECMO configuration, LV may fail to unload, causing progressive LV distension. In such cases, our strategy consisted of a prompt implantation of CentriMag^®^ eVAD (Figure 2), with respect to other decompression strategies which we rarely adopt. The aggressive management is justified by the restoration of a physiological circulatory pattern on *full-flow* support and the complete unloading of all cardiac chambers, even before the onset of LV distension, which impacts seriously on prognosis. In this view, thanks to our initial experience with this strategy, we subsequently performed direct implantations of the CentriMag^®^ eVAD, in those patients who were known to have very low myocardial contractility and where the necessity of prolonged *full-flow* support was foreseen.

We collected the daily percentage of maximal theoretical flow (e.g., percentage ratio between ongoing ECLS flow and maximal theoretical flow) and we then calculated the mean of this ratio during the entire support period (named *mean percentage of theoretical flow support*). For the purposes of analysis, we named the lowest, middle, and highest percentiles *low-, medium- and high-flow*, respectively.

If the patient remained stable (either in case of ECMO or CentriMag^®^ eVADs support) the weaning protocol started. After obtaining hemodynamic stabilization and improvement of organs’ function, ECLS support was progressively decreased up to 1 L/minute. Standard management involved serial echocardiograms, the persistence of satisfactory hemodynamics and organ function, and low-to-medium dose inotropic support. If the patient failed the weaning trial, the recovery of the myocardium was unlikely and the patient was given end-stage heart failure surgery treatment (heart transplantation or intracorporeal VAD).

### 2.5. Anticoagulation Management

Before cannulation, a heparin bolus of 70 U/Kg was administered. After this step, we performed activated partial thromboplastin time (aPTT), international normalized ratio, and antithrombin assays 4 times per day; platelet counts, fibrinogen, and d-dimer assays were conducted once daily.

Patients were kept anticoagulated with unfractionated heparin titrated to maintain an aPTT in the range of 50–60 s. Less stringent anticoagulation was needed with the CentriMag^®^ eVAD when the oxygenator was removed (target aPTT 45–50 s).

### 2.6. Data Analysis

Data are summarized as mean (SD) or median (IQR) in case of quantitative variables, as counts and percentages in case of categorical variables. The normality of quantitative variables was checked with the Shapiro–Wilk test. Quantitative variables were compared across groups with the independent *t*-test and the Mann–Whitney test, as appropriate, and categorical variables with chi-squared test and Fisher’s exact test. The overall survival was estimated according to the Kaplan–Meier method. Differences in survival rates were tested with the log-rank test. Logistic backward stepwise regression models were applied to assess variables related to in-hospital mortality. Cox-regression models were applied to assess variables related to overall mortality; the assumption of proportional hazards was checked by graphical methods. Statistical significance was set at *p* < 0.05. All statistical analyses were performed using SPSS 23.0 (IBM Corporation, Armonk, NY, USA).

## 3. Results

### 3.1. Patients

The preoperative characteristics of the population are summarized in Table 1. A total of 212 patients (162 male, 76%) with a mean age of 53.75 ± 14.61 years were supported with ECLS at our center.

Among the etiologies, acute coronary syndromes were treated more frequently with ECMO alone rather than with CentriMag^®^ eVAD (50% vs. 30%, *p* = 0.012). On the contrary, fulminant myocarditis and idiopathic dilated cardiomyopathies often underwent eVAD implantation (3% vs. 12%, *p* = 0.021 and 19% vs. 36%, *p* = 0.001, respectively), either as an upgrade or as a primary approach. Considering the type of ventricular failure (isolated LV failure vs. biventricular failure), we reported a trend towards a more compromised population in Group 2, where, although not statistically significant, biventricular failure was more common (49% vs. 36%, *p* = 0.073).

No differences were identified among the groups regarding preoperative organ dysfunction and comorbidities.

ECLS specifications are listed in Table 2 and Figure 1, whilst complications during ECLS support are outlined in Table 3. ECMO flows were significantly lower than CentriMag^®^ eVAD ones: in Group 1, the mean percentage of theoretical flow support was 61.21 ± 16.01%, whereas in Group 2 it was 79.49 ± 18.42% (*p* = 0.001).

Patients were supported with ECLS for a mean duration of 10.29 ± 9.66 days, with the longest duration being 46 days. In Group 1, the mean duration of support was 8.37 ± 8.43 days, while in Group 2 it was 14.25 ± 10.84 days (*p* = 0.001).

The 30-day survival was 65%, whilst the rate of patients discharged from the hospital was 53%. The estimated 1-year overall survival after ECLS was 45.7 ± 3.5%. The estimated 1-year survival of discharged patients was 84.1 ± 3.5%.

### 3.2. Flow Patterns

To understand the role of physiological circulation patterns with *high-flow* support between ECMO and CentriMag^®^ eVAD configurations, we performed a comparison through regression models.

Stratifying the ECLS population according to Groups 1 and 2, the logistic regression model revealed that the mean percentage of theoretic flow was a significant predictor of in-hospital mortality only for Group 1 (Group 1: OR 38.62, CI 95% 3.94–378.82; Group 2: OR 10.95, CI 95% 0.66–181.80).

Moreover, the Cox regression model revealed that the mean percentage of theoretical flow was a significant predictor of overall mortality only for Group 1 (Group 1: HR 11.36, CI 95% 2.19–44.20; Group 2: HR 1.48, CI 95% 0.32–6.80).

**Figure 3:**
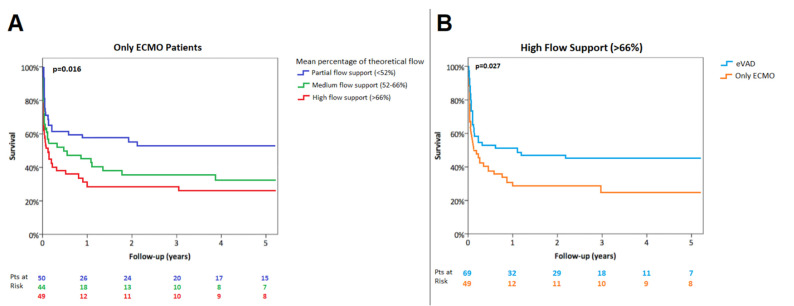
Kaplan–Meier survival according to ECMO flow support (**A**) and with high-flow ECLS configuration (**B**).

Only-ECMO supported patients requiring *high-flow* (>66%, [66th percentile]) were associated with lower in-hospital survival as well as estimated 1-year survival compared with those with a *low-flow* support (<52%, [33rd percentile]) (in-hospital survival 37% vs. 68%, *p* = 0.008; 1-year survival, 28.9 ± 6.8% vs. 57.7 ± 7.0%, *p* = 0.016) (Figure 3A).

In the setting of *high-flow* mechanical support (mean percentage of theoretical flow >66%), in-hospital survival and estimated 1-year survival were higher in patients treated with CentriMag^®^ eVAD in comparison with only ECMO support (in-hospital survival 55% vs. 37%, *p* = 0.037; 1-year survival 50.7 ± 6.0% vs. 28.9 ± 6.8%, *p* = 0.027) (Figure 3B). Clinical outcomes according to ECLS support are summarized in Table 4.

## 4. Discussion

The aim of this study was to evaluate the impact of the ECLS configuration on the outcome of patients in primary cardiogenic shock, according to the needed flow support. Patients were divided into groups based on the configuration of ECLS support, with comparable pre-implantation multi-organ function conditions. We found that a *high-flow* ECMO support had worse in-hospital and long-term survival than *low-flow* support, and in the setting of patients needing high-flow support an eVAD configuration is advisable to improve outcomes.

ECMO represents one of the most widely used temporary mechanical circulatory support modalities due to costs, ease and rapidity of cannulation, and the ability to provide biventricular and respiratory support [2,3]. It is, therefore, the preferred choice in the setting of a rapid deteriorating cardiac function, or even cardiac arrest.

However, a common drawback of ECMO is the associated increase in left ventricular afterload [4,10]. This happens especially when *full-flow* support is needed and the residual myocardial function is not able to open the aortic valve at every cardiac cycle, resulting in an incomplete left ventricle unloading. Increased left ventricular afterload can have deleterious effects including myocardial ischemia, delayed ventricular recovery, pulmonary edema, thrombotic events, and eventually multi-organ dysfunction [11].

Several left ventricular unloading strategies have been described [6,12]. However, there is a lack of evidence about the relationship between the needed percentage of flow support during ECLS and LV unloading. In the setting of ECLS, we believe that the optimal management should be divided into two clinical scenarios according to the mean flow support: *partial-flow* and *full-flow* support.

The first scenario, *partial-flow* support, was well characterized by a computational model of Caruso et al. during ECMO [13]. In this study, the authors highlighted that the pulse contour is higher and more similar to the physiological pattern when there is a percentage of blood flow provided directly by the heart. Furthermore, the ascending aorta is characterized by blood stagnation during total support, both with IABP-on and IABP-off, whereas in the case of double perfusion (*partial support* with ECMO plus residual heart function) the flow is always orderly. Our results are consistent with these findings, showing lower mortality in patients treated with ECMO alone when *partial-flow* support was required. In these patients, the ECMO strategy is safe and effective and it is the only necessary treatment.

The second scenario is the *full-flow* support. Mortality increased when *high-flow* was required during ECMO support. In these cases, our policy is to shift towards eVAD Centrimag^®^ for left ventricular unloading, especially whenever the residual myocardial contractility is extremely depressed and with a biventricular involvement [14]. This is a common finding in fulminant myocarditis or end-stage dilated cardiomyopathy, while it is less frequent after acute coronary syndromes, explaining the different rate of CentriMag^®^ eVAD implantation according to etiology in our series [15,16]. On the other hand, we previously reported that survival of patients treated with ECLS is not influenced by the acute versus chronic etiology of primary cardiogenic shock [17]. Moreover, a recent sub-analysis of the EUROMACS registry revealed that, after propensity-score matching, ischemic cardiomyopathy and idiopathic cardiomyopathy display similar 30-day-mortality and long-term survival, supporting the hypothesis that comorbidities are the most likely determinant of the patient’s prognosis [18]. In our cohort, preoperative variables of organ dysfunction were all comparable between study groups, as well as the critical presentation of cardiogenic shock with circulatory arrest (Table 1). For this reason, we speculate that the different type of ECLS (ECMO vs. eVAD) might play an active role in determining patient outcomes.

Among the key factors to consider for ECLS upgrade from ECMO, besides flow support, is the type of used device (IABP vs. Impella vs. apical venting vs. eVAD) [6,7,19].

Results of IABP insertion during ECMO support are conflicting and with dubious effectiveness [20,21,22,23]. Direct apical venting can provide *full-flow* support but without a physiological circulation pattern in the pulmonary vessels, leading to altered lung function. Unfortunately, only few reports provide a documented experience with apical venting: Centofanti et al. described interesting results with a median ECLS support of 11.5 days [24]. However, even in that study, the configuration was switched from ECMO with apical venting to eVAD in some patients, by eliminating the venous cannula from the system. In our experience, when a patient is kept on full-flow ECMO support, they are monitored daily to assess the presence of a residual arterial pulsatility, opening of the aortic valve, and to exclude the occurrence of LV distension. If these conditions are not satisfied, we prefer to upgrade to eVAD support, rather than adopting other unloading solutions.

A wide-ranging strategy of unloading is the percutaneous implantation of short-term ventricular assist devices, the most used of which is the Impella^®^ system (Abiomed, Danvers, MA, USA) [25,26]. Pappalardo et al. reported that EC-PELLA (ECMO associated with Impella^®^) had lower in-hospital mortality in comparison with ECMO alone [26]. However, they described a high rate of hemolysis (76%) and major bleeding (38%) with EC-PELLA. Moreover, the median mechanical support time was fairly short (6.2 days), probably due to time-related support complications. Other well-known Achilles heels of Impella^®^ are partial support (depending on the design, e.g., Impella^®^ 2.5, CP or 5), system dislodgement, and vascular complications. Pappalardo et al. also reported a case of percutaneous biventricular support with Impella^®^ (Bi-PELLA): however, the maximum flow was 3.6 L/min with nearly-ceiling rotational pumps speed and documented hemolysis [27].

In our experience, the CentriMag^®^ eVAD configuration has proved to be a versatile, effective and reliable system for ventricular assistance and it provided full circulatory support (up to 10 L/min) with complete unloading of the heart and a physiological flow pattern simply detectable on chest X-ray. Published reports on CentriMag^®^ eVAD demonstrate good results in terms of survival, but without a comparison with other ECLS configurations [8,28,29]. John et al. reported a median support time of 13 days, with a low incidence of device-related hemolysis (5%) and bleeding (5%) [28]. Zeriouh et al. described a median support time of 30 days and an incidence of major bleeding of 35%, while the widest series of Takayama et al. highlighted a median support time of 20 days with a rate of major bleeding of 33% [8,29]. Our findings with CentriMag^®^ eVAD showed a median time support of 14.25 days (the longest was 54 days), consequently more than double with respect to EC-PELLA results, with lower rates of hemolysis (29%) and major bleeding (16%).

The implantation procedure of CentriMag^®^ eVAD is not technically challenging and does not require the use of cardiopulmonary bypass per se. One disadvantage may be the trauma due to the surgical access [30]. However, compared to other short-term mechanical circulatory support therapies, CentriMag^®^ eVAD is less susceptible to clot formation and, as a consequence, anticoagulation is less stringent [31]. If needed, the beginning of anticoagulation can be withheld for 48 to 72 h and this gives time for bleeding to resolve. The high hemocompatibility could explain the lower incidence of major bleeding in our series, the lower rate of hemolysis, and the longer time of mechanical support without increase in complications compared to percutaneous strategies.

VA-ECMO is an optimal and versatile life-saving option in our experience as long as its duration is limited and *partial-flow* support is considered. In particular, VA-ECMO support beyond 9 days shows a less favorable outcome with increased mortality rate [17].

Conversely, although more invasive, CentriMag^®^ eVAD configuration allows a longer mechanical support free from time-related complications. Moreover, it is an all-around tool in the setting of *high-flow* support which provides (1) full unloading of both ventricles, (2) high flow up to 10 L/min, and (3) physiological (systemic and pulmonary) circulation patterns adjustable to different clinical conditions (Table 5).

With the aim to minimize the disadvantages of its surgical implantation, a less invasive perspective might be achieved by the association of a left CentriMag^®^ eVAD, implanted throughout a left mini-thoracotomy, with a percutaneous double lumen cannula via the jugular vein to achieve a right ventricular assistance, for example with the Protek-Duo^®^ device. We speculate that this strategy, in selected patients, has the potential to further improve the clinical outcomes of the eVAD configuration.

## 5. Limitations

This study presents some limitations: first, the different distribution of etiologies in the two groups likely influenced the strategy of choice. Furthermore, due to ease and rapidity of implantation, ECMO is always the preferred choice in emergent cases, which are known to experience a worse prognosis. Nevertheless, distribution of isolated LV vs. biventricular failure, clinical conditions, and organ dysfunction variables at implantation were similar between groups (Table 1), allowing a satisfactory comparison. Furthermore, due to the growing experience with the eVAD configuration, in recent years we have also managed to implant this device in urgent cases, with very poor residual ventricular function, minimizing this selection bias.

Second, a subgroup of eVAD patients started support on ECMO and were later upgraded. However, this is in line with our aim to measure the effect of LV unloading in a *high-flow* setting.

Third, we present our experience over nearly a decade, which saw some technical developments, and possibly some minor changes to the system, but allowed us to collect one of the largest series so far.

Lastly, since our centre’s preferred strategy to vent the LV is eVAD, we lacked the opportunity to directly compare our approach with other methods for LV unloading, such as LV apical venting, Impella, or other devices.

## 6. Conclusions

ECLS should be considered mainly as a rescue approach for the treatment of refractory cardiogenic shock. We propose a patient-specific strategy based on the degree of mechanical support required, identifying two scenarios. In the context of deteriorating cardiac function requiring *partial-flow* support, VA-ECMO remains a balanced support strategy in terms of ease of implantation and low invasiveness. When *full-flow* support is necessary, CentriMag^®^ eVAD represents a safer and more effective option that provides complete cardiac unloading, high flow support, physiological circulatory patterns, and duration of support.

## Figures and Tables

**Table 1:** Pre-implantation characteristics.

	Overall	Group 1	Group 2	*p*-Value
	(n = 212)	(n = 143)	(n = 69)	
Age (years)	53.75 ± 14.61	54.87 ± 15.02	51.42 ± 13.54	0.108
Male	162 (76%)	115 (80%)	47 (68%)	0.058
BSA (m^2^)	1.88 ± 0.28	1.89 ± 0.31	1.85 ± 0.21	0.258
Etiology				
Acute coronary syndrome	92 (43%)	71 (50%)	21 (30%)	**0.012**
Myocarditis	12 (6%)	4 (3%)	8 (12%)	**0.021**
Pulmonary embolism	5 (2%)	4 (3%)	1 (1%)	0.475
Ischemic cardiomyopathy	34 (16%)	20 (14%)	14 (20%)	0.318
Dilated cardiomyopathy	52 (25%)	27 (19%)	25 (36%)	**0.010**
Congenital heart disease	2 (1%)	2 (1%)	0 (0%)	0.454
Other	17 (8%)	15 (10%)	2 (3%)	0.063
Isolated LV failure	126 (59%)	91 (64%)	35 (51%)	0.073
Biventricular failure	86 (41%)	52 (36%)	34 (49%)	0.073
Mechanical ventilation	180 (85%)	118 (83%)	62 (90%)	0.219
Renal failure	89 (42%)	62 (43%)	27 (39%)	0.656
Continuous hemofiltration	19 (9%)	12 (8%)	7 (10%)	0.797
Hepatic failure	36 (17%)	25 (17%)	11 (16%)	0.847
MELD score	13.29 ± 7.60	13.56 ± 7.48	12.78 ± 7.84	0.490
Number of inotropes	1.45 ± 1.15	1.49 ± 1.12	1.37 ± 1.23	0.513
Intra-aortic balloon pump	63 (30%)	46 (32%)	17 (25%)	0.336
Cardio-pulmonary resuscitation	47 (22%)	33 (23%)	14 (20%)	0.647

**Table 2:** ECLS specifications.

	Overall	Group 1	Group 2	*p*-Value
	(n = 212)	(n = 143)	(n = 69)	
ECMO duration (days)	7.43 ± 7.72	8.37 ± 8.43	4.55 ± 3.73	**0.003**
ECMO mean flow/theoretic flow (%)	64.02 ± 18.33	61.21 ± 16.01	72.21 ± 22.05	**0.002**
Left eVAD implantation	35 (17%)	-	35 (51%)	-
Biventricular eVAD implantation	34 (16%)	-	34 (49%)	-
eVAD duration (days)	11.12 ± 10.35	-	11.12 ± 10.35	-
eVAD mean flow/theoretic flow (%)	79.49 ± 18.42	-	79.49 ± 18.42	-
Total ECLS duration (days)	10.29 ± 9.66	8.37 ± 8.43	14.25 ± 10.84	**0.001**
Total ECLS mean flow/theoretic flow (%)	67.19 ± 18.87	61.21 ± 16.01	79.49 ± 18.42	**0.001**

**Table 3:** Major complications during ECLS.

	Overall	Group 1	Group 2	*p*-Value
	(n = 212)	(n = 143)	(n = 69)	
Neurological event	35 (17%)	27 (19%)	9 (13%)	0.114
Sepsis	23 (11%)	14 (10%)	9 (13%)	0.471
Renal failure	150 (71%)	97 (68%)	53 (77%)	0.178
Continuous hemofiltration	77 (36%)	45 (31%)	28 (41%)	0.057
Hepatic failure	91 (43%)	59 (41%)	32 (46%)	0.481
ARDS	23 (11%)	11 (8%)	6 (9%)	0.367
Hemolysis	52 (25%)	32 (22%)	20 (29%)	0.234
Major bleeding	31 (15%)	20 (14%)	11 (16%)	0.532

**Table 4:** Clinical outcomes.

	Overall (n = 212)	*Partial-Flow* ECMO (n = 50)	*Medium-Flow* ECMO (n = 44)	*High-Flow* ECMO (n = 49)	*High-Flow* eVAD (n = 69)	*p*-Value (All Subgroups Comparisons)	*p*-Value (*High-Flow* ECMO vs. *High-Flow* eVAD)
Mortality during ECLS	51 (24%)	4 (8%)	12 (27%)	20 (41%)	15 (22%)	**0.002**	**0.021**
In-hospital mortality	99 (47%)	16 (32%)	21 (48%)	31 (63%)	31 (45%)	**0.020**	**0.037**
Myocardial recovery	59 (28%)	31 (62%)	13 (30%)	7 (14%)	8 (12%)	**0.001**	0.435
Heart transplantation after ECLS	45 (21%)	8 (16%)	11 (25%)	5 (10%)	21 (30%)	**0.041**	**0.007**
LVAD implantation after ECLS	36 (17%)	7 (14%)	5 (11%)	5 (10%)	19 (28%)	**0.039**	**0.017**
TAH implantation after ECLS	4 (2%)	0 (0%)	0 (0%)	4 (8%)	0 (0%)	**0.004**	**0.028**
Conventional surgery after ECLS	10 (5%)	3 (6%)	1 (2%)	6 (12%)	0 (0%)	**0.016**	**0.004**

**Table 5:** LV unloading strategies.

Device	LV Unloading	*High-Flow* Support	Physiological Flow	Long-Term Support
ECMO + IABP	↑	-	-	-
ECMELLA	↑↑	↑	-	-
ECMO + Apical Vent	↑↑↑	↑↑	-	↑
BiPELLA	↑↑	↑↑	↑↑	-
CentriMag^®^ eVAD	↑↑↑	↑↑↑	↑↑↑	↑↑↑

## Data Availability

Data available on request to the corresponding author.

## Abbreviations

ECMO	extracorporeal membrane oxygenation
VA-ECMO	venous-arterial ECMO
eVAD	extracorporeal ventricular assistance device
ECLS	extracorporeal life support
LV	left ventricle
IABP	intra-aortic balloon pump
BSA	body surface area
aPTT	activated partial thromboplastin time
TAH	total artificial heart
